# BioClay nanosheets infused with GA3 ameliorate the combined stress of hexachlorobenzene and temperature extremes in *Brassica alboglabra* plants

**DOI:** 10.3389/fpls.2022.964041

**Published:** 2022-10-06

**Authors:** Aqeel Ahmad, Tanveer Alam Khan, Sharoon Shahzad, Sami Ullah, Iqra Shahzadi, Aamir Ali, Waheed Akram, Nasim Ahmad Yasin, Mohammad Yusuf

**Affiliations:** ^1^Institute of Geographic Sciences and Natural Resources Research, Chinese Academy of Sciences (CAS), Beijing, China; ^2^Department of Botany, Aligarh Muslim University, Aligarh, Uttar Pradesh, India; ^3^Incharge Medical Officer, Basic Health Unit Munday Key District Kasur, Kasur, Pakistan; ^4^Department of Forestry, College of Agriculture, University of Sargodha, Sargodha, Pakistan; ^5^School of Resource and Environmental Science, Wuhan University, Wuhan, China; ^6^Department of Botany, University of Sargodha, Sargodha, Pakistan; ^7^Department of Plant Pathology, Institute of Agricultural Sciences, University of the Punjab, Lahore, Punjab, Pakistan; ^8^Senior Superintendent Gardens, RO-II Wing, University of the Punjab, Lahore, Punjab, Pakistan; ^9^Department of Biology, College of Science, United Arab Emirates University, Al Ain, United Arab Emirates

**Keywords:** BioClay, *Brassica alboglabra*, HCB, stress, temperature extremes

## Abstract

Environmental pollutants and climate change are the major cause of abiotic stresses. Hexachlorobenzene (HCB) is an airborne and aero-disseminated persistent organic pollutants (POP) molecule causing severe health issues in humans, and temperature extremes and HCB in combination severely affect the growth and yield of crop plants around the globe. The higher HCB uptake and accumulation by edible plants ultimately damage human health through the contaminated food chain. Hence, confining the passive absorbance of POPs is a big challenge for researchers to keep the plant products safer for human consumption. BioClay functional layered double hydroxide is an effective tool for the stable delivery of acidic molecules on plant surfaces. The current study utilized gibberellic acid (GA_3_) impregnated BioClay (BioClay_*GA*_) to alleviate abiotic stress in *Brassica alboglabra* plants. Application of BioClay_*GA*_ mitigated the deleterious effects of HCB besides extreme temperature stress in *B. alboglabra* plants. BioClay_*GA*_ significantly restricted HCB uptake and accumulation in applied plants through increasing the avoidance efficacy (AE) up to 377.61%. Moreover, the exogenously applied GA_3_ and BioClay_*GA*_ successfully improved the antioxidative system, physiochemical parameters and growth of stressed *B. alboglabra* plants. Consequently, the combined application of BioClay and GA_3_ can efficiently alleviate low-temperature stress, heat stress, and HCB toxicity.

## Introduction

Persistent organic pollutants (POPs) are one of the major environmental obstacles, which have been gradually intensified during the period of massive industrialization (Ahmad et al., 2021). Now, they are not only harming the environment, but their health hazards have crossed alarming levels. Besides the contaminating nature they are also characterized as quick and passively transmitting pollutants in the environment even in the absence of any anthropogenic activity. POPs possess the tendency to halt in the air, resist environmental degradation, and accumulate in the fat-rich tissues (of plants and/or humans) by passing through the phospholipid membranes (Ibrahim et al., 2016; Berntssen et al., 2017). Considering the health hazards of POPs highlights the disturbed reproductive system, disordered metabolism, and carcinogenicity (Wang et al., 2008). Among POPs, hexachlorobenzene (HCB) is highlighted as a fairly volatile compound with long-range air transportation and lipophilic properties. Although, measures have been taken to control the emission of HCB (e.g., banned in the agriculture sector), some industrial and environmental factors are continuously contributing tonnes of HCB into the air annually. Enlisting the key sources of HCB emission ranks smelting of metals, bleaching of paper pulp, production of cement, impregnation of wood, incineration of solid waste and sewage sludge, etc. Besides, HCB is released as a byproduct of all the chemical industries dealing with or producing chlorinated chemicals/liquids. Vinyl chloride, pentachloronitrobenzene, trichloroethylene, pentachlorophenol, tetrachloroisophthalonitrile, picloram, propazine, mirex, and hundreds of other chemicals add significant HCB quantities into the environment during their synthesis or incineration. HCB is readily absorbed in aerial plant parts, translocated between different tissues, and finally preserved in the lipid-rich areas. High HCB contents have been recorded in plants (e.g., pumpkins) from where they entered into the food chain and traveled in the whole food chain, including fish flesh and fish oil (Berntssen et al., 2017). HCB highly tends to be absorbed in the human body with direct damage to the liver and possesses both acute and subchronic toxicity. It can disrupt the function of glycolysis enzymes and slow down the metabolism (Mazzetti et al., 2004). Whereas, genotoxic effects have also been proven in case of prolonged or frequent exposure to HCB (Ezendam et al., 2004). Most importantly, the pollutant is classified among the carcinogens of group 2B, making its complete avoidance necessarily important, which seems impossible if the pollutant is a part of our daily diet. Limiting HCB contents in agri-food products can be of great assistance in this task. Therefore, some strategies must be developed to control aero-concentration and bioaccumulation of HCB in agricultural crops, especially leafy vegetables, e.g., *Brassica alboglabra*) which are directly consumed by humans.

Gibberellic Acid (GA_3_) is the parent molecule of hundreds of gibberellins (GAs) classified as plant growth regulators. Studies have reported multi-directional regulation of plant responses under the influence of GAs. GA_3_ is a tetracyclic diterpenoid molecule bearing a carboxy group, two hydroxyl groups, and a lactone ring. Researchers have utilized GA_3_ and its derivatives to control seed germination, plant growth (vigor and height), flowering time, leaf senescence, etc. Considering its diverse physiological effects in plants, GA_3_ was selected to assess its ability to reduce HCB absorption from the surrounding air. This is the first time the molecule has been tested to limit the absorption and translocation of a POP. However, kinetics models show that the half-life of GA_3_ is gradually lowered at a temperature higher than 20°C. The half-life of GA_3_ is only 2 h at 50°C, which is occasionally achieved during the summers of hot regions (Palmer, 1974). However, providing continuous and minute delivery of GA_3_ to plant cells is a necessary task, rather than spraying larger currents of the growth regulator.

Ever-increasing environmental temperature due to global warming makes the situation more challenging for GA_3_ delivery. High-temperature fluctuations not only dissipate GA_3_, but also abnormalize plant development. Temperature is one of the most important phenological stimulators, and induces diverse physiological responses in the plants depending upon their developmental stages (Bahuguna and Jagadish, 2015; Ahmad et al., 2018). Small fluctuations in the temperature cause serious disturbances in the phenological cycle of the crops, which could be a food security threat if the plants are already under stress conditions (Khan et al., 2018; Zaheer et al., 2018). The temperature has been well-documented for its role in regulating almost all the physiological and developmental processes, including stomatal conductivity, water contents, photosynthesis, plant growth, fruit senescence, fruit ripening, etc. (Zandalinas et al., 2018; Farooq et al., 2019). Increased temperature impacts the enzymes’ activities and drives the plants under stress by disturbing evapotranspiration rates (Lamaoui et al., 2018). The modulated enzyme activities become a leading cause of altered protein concentrations, leaf water contents, sugar contents, osmoprotectants, and oxidative enzymes, e.g., superoxide dismutase (*SOD*), ascorbate peroxidase (*APX*), and peroxidase (*POD*) (Sattar et al., 2020). Not only the high temperatures but the low temperature also abnormalizes the plant’s growth, antioxidants activity, chlorophyll contents, reactive oxygen species (ROS), rate of photosynthesis, and several other physiological factors.

*Brassica* is an oil seed-producing plant that is famous for its consumption as a leafy vegetable and animal feed. Growing such an important crop in an HCB polluted environment causes the pollutant to be absorbed, translocated, and preserved in the lipid-rich plant parts, e.g., seeds. It is very important to restrict the entry of the pollutant into the plant foliage to keep the plant free of HCB contents. On the other side, research on foliar uptake of organic POPs and the way to control their accumulations is dearth studied.

To make plants withstand temperature fluctuations and to release stable and slow release of GA_3_ on the plant surface, we selected BioClay as a delivery agent. BioClay is a sheet-like clay nanoparticle with functional layered double hydroxide clay nanosheets that precipitates naturally in saline water bodies or through the weathering of basalts. BioClay nanosheets are a family of inorganic layered materials (Ram Reddy et al., 2006, 2008; Xu et al., 2006), which can be synthesized using a cost-effective protocol (Xu et al., 2006). The main features of BioClay are to keep acidic molecules stable and release slowly on the plant surfaces. The BioClay can be topically applied to plants for increased plant growth and defense parameters (Mitter et al., 2017). Therefore, the present research was designed to investigate the role of GA_3_-coated BioClay (BioClay_*GA*_) in alleviating multiple stress (HCB and temperature) conditions in Kale plants and their joint effort to control the bioaccumulation of the toxic organic contaminant. A number of positive physiological impacts have already been documented against the BioClay application (Fletcher et al., 2020). Henceforth, it was hypothesized that the combined application of BioClay with GA_3_ might have beneficial effects on plant stress alleviation and growth improvement. According to our information, no investigation has been executed to elucidate the role of GA_3_ or/and BioClay in alleviating the effects of multiple environmental stressors. Therefore, during the current study, the potential of GA_3_ impregnated BioClay was first time evaluated to alleviate HCB and temperature stress in *B. alboglabra* plants. This study elucidates the role of BioClay_*GA*_ on growth, antioxidative system, and gaseous exchange attributes of *B. alboglabra* plants under HCB and temperature stress.

## Materials and methods

### Plant material and growth conditions

*Brassica* seeds were submerged in potassium permanganate (0.1%) for 15 min to ensure their surface sterilization and then washed three times with sterilized deionized water to completely remove any traces of the sterilizing agent. Bedding two Whatman filter papers prepared glass jars, and standard Davtyan (10 mL, 0.75 N) nutrient solution was flooded to maintain the moisture. Then, 10 sterilized seeds were placed into each jar under very low light and placed (Ahmad and Ashraf, 2016). All the jars were exposed to dark and 2°C temperatures for five days in a plant growth chamber. Five-day-old seedlings were transferred to soil pots at the rate of two seedlings per pot, while the pots contained one kilogram of UQ23 pot soil.

### BioClay_*GA*_ synthesis

The method of Mitter et al. (2017) was strictly followed to prepare BioClay nanosheets. The process included non-aqueous precipitation, heat treatment, purification step, and dispersion in water. The complete procedure yielded an average particle size of 45 nm, which was analyzed by a Nanosizer, Nano ZS instrument (Malvern Instruments) to obtain the Z average size and PdI. The chemical composition and crystal structure were verified by powder XRD with five BioClay samples (Rigaku Miniflex X-Ray diffractometer), Fourier transforms infrared spectroscopy (Nicolet 6700 FT-IR; Thermo Electron Corporation) with attenuated total reflection mode (Xu et al., 2006) and imaged by JEOL transmission Electron-microscope, JSM-2010.

### Loading of GA_3_

To define optimal and complete loading of respective GA_3_ into BioClay nanosheets, the ratio of GA_3_ to BioClay was adjusted to 1:10. A mixture of GA_3_ 10 mL and BioClay 100 mL was prepared and incubated at room temperature for 30 min with continuous agitation of 120 rpm. Quantifying the residual GA3 was done using Sun et al.’s standard method (Sun et al., 2020). HPLC system equipped with ZORBAX-Eclipse XDB-C18 column (4.6 × 250 mm, 5 μm, Agilent) separated the free GA_3_ molecules in an aqueous mobile phase of 40% methanol flowing at the rate of 0.4 mL min^–1^. Samples of 10 μL were separately processed at 40°C, and absorbance readings were recorded at 210 nm. The stable release of GA_3_ was also ensured at three different incubation temperatures 0, 25, and 50°C for a period of sixty by using the method described above.

### Treatment application

GA_3_ (C_19_H_22_O_6_) was purchased from RPI Research Products International, CAS # 77-06-5. The growth regulator was initially dissolved in ethanol to prepare a stock concentration of (0.5 mM L^–1^), and then diluted with distilled sterilized water to attain a concentration of 5 μM L^–1^ as a working concentration recommended by Miao et al. (2017). Similarly, the BioClay_*GA*_ solution was prepared to get the final working concentration of 0.5 mM L^–1^ for GA_3_. However, the concentration of BioClay was 1 g L^–1^. The third treatment of BioClay nanoparticles was also prepared (1 g L^–1^). Each seedling received 0.5 mL of the respective spray treatment.

To determine the HCB uptake pathway in the *Brassica* plants, specially designed incubation chambers (ICs) were introduced in the experimental design. The ICs consisted of glass materials, and the design and manufacturing pattern was adopted from a previous study (Zhu et al., 2016). The design had two separate air inlets, among which one was used for HCB and the second was for air. Both inlets had electrically controlled air pumps wired and connected with polytetrafluoroethylene hoses. The ICs had their own temperature sensing and regulatory system, while an air sampling window was also installed in every IC to collect a sample without significantly disturbing the experimental setup. Fine silica sand with a particle size of 150-380 μm was used to cover the potting soil to avoid direct contact between the HCB-contaminated air and the potting soil (Zhu et al., 2020). The HCB concentration in the air was adjusted to 1,000 mg m^–3^ as derived from its minimum toxic concentration of 582.4 mg m^–3^ and kept constant throughout the incubation period (Jarrell et al., 2002). In order to compensate for the time course losses of particulate on the plant surface, the POP dust was sprayed twice a week. Afterward, the growth conditions in ICs were maintained as relative humidity (70-76%), photoperiod (16 h), and light (500-550 mmol m^–2^ s^–1^). Five biological replications were used for each treatment.

### Temperature treatment

*Brassica* seedlings were treated with a temperature shock of 0, 5, 15, 25 (experimental temperature), 35, 45, and 50°C for 2 h during the light period (Jiang et al., 2018). By following this design, the experiment contained a total of 124 treatments, including one control treatment (Supplementary Table 1). The entire experiment was incubated until seed production. Plant tissues (roots, shoots, and leaves) were randomly sampled at the age of 8 weeks.

### Breakdown of BioClay and release of GA_3_

Breakdown of BioClay and release of GA_3_ were necessary to expose plants to GA_3_ treatment. To detect the degradation, 100 μL of BioClay suspension was dispensed in 20 droplets on detached *Brassica alboglabra* leaves (four replicates) with a similar surface area. Petri-plates were bedded with moistened filter papers, and the collected leaf samples were placed on them. The leaf petioles were immersed in Murashige-Skoog (MS) basal media (SigmaAldrich) in small glass vials taped to the dish. The Petri-plates were incubated in an incubation chamber with relative humidity (95%), CO_2_ (5%), and temperature (27°C). Leaf samples were collected after the interval of 1, 3, 5, and 7 days post-application of BioClay. The collected leaf samples were rinsed with a 10 mL aqueous solution of HNO_3_ (2%) and ethanol (3%). The rinse solutions were collected and analyzed through inductively coupled plasma-optical emission spectrometry (ICP-OES) to determine relative magnesium and aluminium ion concentrations. Four identical samples were processed separately. One sample was maintained outside the chamber at room temperature under normal atmospheric conditions (0.045% CO_2_). After 7 days of incubation, the tubes were centrifuged at 12,000 rpm for 15 min. The supernatant was pas processed at HPLC to detect the amount of free GA_3_ contents (Sun et al., 2020). HPLC system equipped with ZORBAX-Eclipse XDB-C18 column (4.6 × 250 mm, 5 μm, Agilent) separated the free GA_3_ molecules in an aqueous mobile phase of 40% methanol flowing at the rate of 0.4 mL min^–1^. Samples of 10 μL were separately processed at 40°C, and absorbance was read at 210 nm.

### Stability of GA_3_ bound to BioClay

To check the stability of GA_3_, the release of the compound was tested at varying temperatures (0-50°C) using the similar HPLC method. Meanwhile, an aqueous GA_3_ solution of identical concentration was taken as a control treatment under similar temperature conditions. The samples were sprayed on *Brassica* leaves, and the method of GA_3_ detection was followed again as described earlier. After an incubation period of 1 month and 2 months, the samples were chromatographically analyzed for GA_3_ contents (Sun et al., 2020).

### Determination of growth, antioxidant, and physiological parameters

After 8 weeks of incubation, all the plants were randomly sampled, and the collected samples were separated into the leaves, roots, and shoots. Each tissue was washed for one minute with the ddH_2_O and dried on blotting paper prior to its storage at −80°C for further analytical use. The plant samples were estimated for their growth parameters (shoot length, root length, shoot fresh mass, root fresh mass, shoot dry mass, root dry mass, SPAD chlorophyll, and leaf area), soluble sugars, net photosynthetic rate (*Pn*), photosynthetic pigments (Chl *a*, Chl *b*), photosystem quenching (*Fv/Fm*), gaseous exchange parameters, H_2_O_2_ contents, leaf osmotic potential, water potential, electrolyte leakage (*EL*), membrane stability index (*MSI*), proline content, lutein, lycopene, β-carotenoids, malondialdehyde (MDA), oxidative and antioxidant enzymatic activities (Carbonic anhydrase (*CBH*), nitrate reductase (*NR*), peroxidase (*POD*), *catalase* (*CAT*), superoxide dismutase (*SOD*), monodehydroascorbate reductase (*MDHAR*), dehydroascorbate reductase (*DHAR*), ascorbate peroxidase (*APX*), glutathione-*S*-transferase (*GST*), glutathione reductase (*GR*), and *glutathione* peroxidases (*GPX*)) by strictly following the standard optimized protocols (Li et al., 2021).

### Determination of hexachlorobenzene in plants

The method of Liu et al. (2013) was adopted to estimate HCB contents in the plants. An accelerated solvent extraction system (ASE 200, Dionex, Sunnyvale, CA) was used to extract and collect chlorobenzenes (hexachlorobenzene and its metabolites) from *Brassica* plants. Each sample (seeds, shoots, and roots) was weighed (5 g) and homogenized with 5 g of diatomaceous earth. Whereas the physical conditions were kept at the temperature (90°C) and pressure (10 MPa). An extraction solvent was prepared by mixing hexane/acetone with the ratio of (3:1, vol/vol) and used to extract the chlorobenzenes. The extract was collected in the glass flask and evaporated under a vacuum at 45°C using a rotary evaporator (Yarong, Shanghai, China) to achieve the final volume of 2 mL of concentrated extracts. A step of solid-phase extraction was employed to clean the extracts by passing through the extraction cartridges containing Na_2_SO_4_ (2 g), and silica gel (1 g). A solvent system of hexane/dichloromethane (15 mL) was used at the ratio of 9:1 (vol/vol), to elute the extracts.

A pre-optimized gas chromatograph (Agilent 6890, Santa Clara, CA) was used to measure the concentrations of HCB and its metabolites. The gas chromatograph was equipped with a DB-5 capillary column (J&W Scientific, Folsom, CA) with the dimension 30-m length × 0.32-mm inside diameter × 0.25-μm film thickness. An HP 7683 auto-sampler (Hewlett Packard, Mississauga, ON) was used to load the sample on the chromatograph, and a ^63^Ni electron capture detector was used to plot the chromatograms. The carrier gas in the experimental operation was inert nitrogen. The temperature of the injector was 240°C, while the temperature of the detector was 290°C. One microliter of each sample was injected into the chromatograph in a splitless mode. The recovery for HCB in seeds, shoots and roots was 98.9, 97.4, and 93.7%, respectively. The sensitivity of the gas chromatograph to detect trace amounts of HCB was 0.5 pg μL^–1^.

### Calculation of mobility, avoidance efficiency and pollutant tolerance index

The translocation factor (*TF*), and avoidance efficiency (*AE*) were used to evaluate the ability of the plants to absorb, translocate, and/or avoid pollutants, according to the following equations (Xiao et al., 2019; Zeng et al., 2019).

(1) *TF* = C_*substance shoot*/_C_*substance leaf*_
(2) *AE* = (C_*substance shoot*_ × M_*shoot*_ × C_*substance*__*leaf*_ × M_*leaf*_)/(C_*substance air*_ × M_*air*_)

where the C_*substance shoot*_, C_*substance leaf*_, and C_*substance air*_ are the contents of HCB (mg⋅kg^–1^) in shoots, roots, and air, respectively. However, the mass of one cubic meter of air was standardized as 1.29 kg during the experimentation.

Pollutant tolerance index (PTI) from plant biomass was calculated by employing the following formula of Deng et al. (2007):

PTI=DW/t⁢r⁢e⁢a⁢t⁢e⁢d⁢p⁢l⁢a⁢n⁢tDW.c⁢o⁢n⁢t⁢r⁢o⁢l

### Statistical analysis

Data were presented in the form of mean ± SE of the five biological replicates. Analysis of variance (ANOVA) was performed by the MS-Excel add-in statistical package DSAASTAT (Onofri, Italy). The significant differences among treatments were determined by Duncan’s Multiple Range Test (DMRT) at *p* ≥ 0.5.

## Results

### Structural estimation of BioClay_*GA*_

**FIGURE 1:**
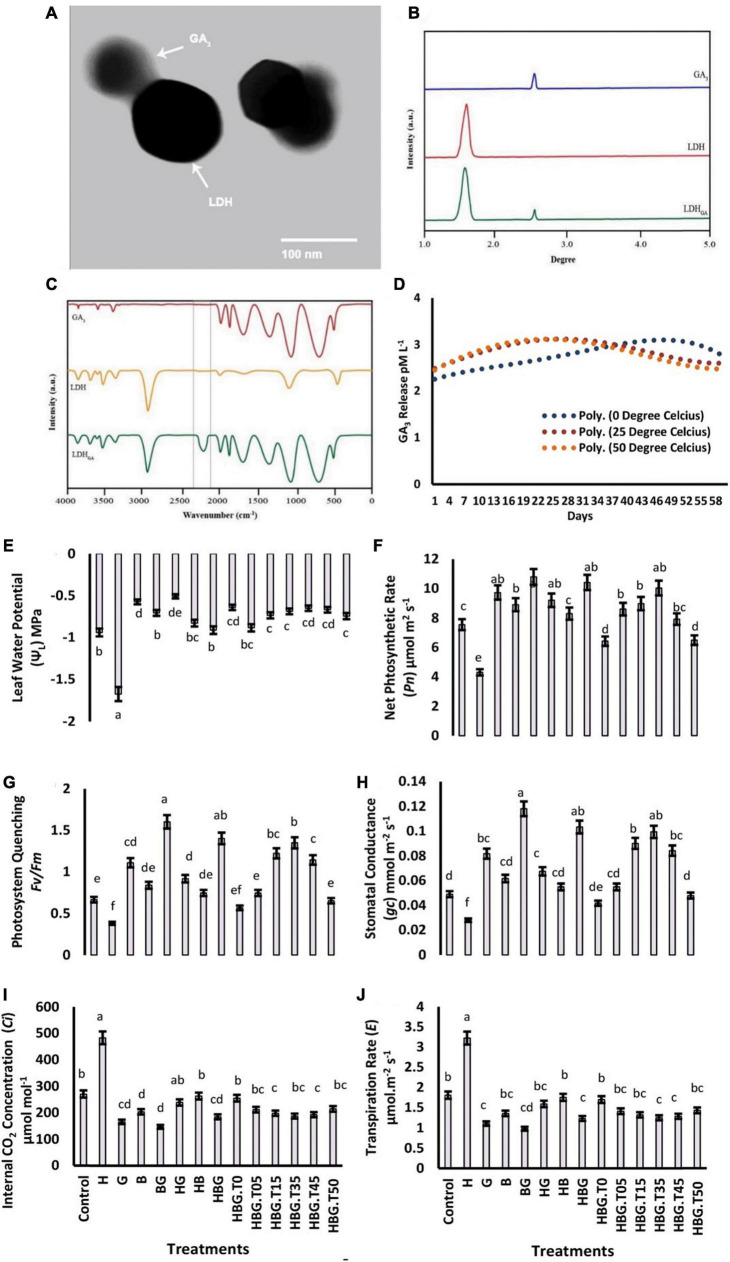
Electron microscopic image of bioclay nanoparticle with impregnated GA_3_ **(A)**. X-ray diffraction analysis-XRD of gibberellic acid-GA_3_, bioclay-LDH, and gibberellic acid impregnated bioclay-LDH_*GA*_ **(B)**. Fourier-transform infrared spectroscopic-FTIR analysis of GA_3_, LDH, and LDH_*GA*_ **(C)**. Time course relesse of GA3 molecules by bioclay nanoparticles at three different temperatures, i.e., (0, 25, and 50 degree celcius **(D)**. Effect of GA_3_ on photosynthesis-related parameters of *Brassica alboglabra* under HCB toxicity, and temperature stress. Leaf relative water content **(E)**, Net photosynthesis rate **(F)**, Maximum quality yield of PS-II **(G)**, Stomatal conductance **(H)**, Internal CO_2_ concentration **(I)**, Transpiration rate **(J)**. Values demonstrate means ± SD (*n* = 5). Different letters indicate a significant difference among the treatments (*P* ≤ 0.05). H = HCB; G = GA_3_, B = Bioclay, T0 = 0°C, T05 = 05°C, T15 = 15°C, T35 = 35°C, T45 = 45°C, T50 = 50°C. All treatments without temperature tags got a continuous incubation temperature of 25°C.

The structural analysis of BioClay_*GA*_ revealed a crystalline nature of the nanosheets with 1.7-3.2 μm width and 80-150 nm thickness. An electron diffraction angle of 45°, and an interfacial angle of 43.4° were recorded. The interplanar lattice spacing, atomic plan spacing, and fringe spacing were measured at 0.0272, 0.35, and 0.384 nm, respectively (Supplementary Table 2). Assays clearly showed the GA_3_ molecules impregnated with BioClay nono sheets. Transmission electron micrograph, FTIR spectrum and XRD analysis collectively revealed a stable adhesion of GA_3_ with BioClay. The time-course release of GA_3_ was observed to be greater at a higher temperature (50°C). However, the rate of GA_3_ release possessed non-significant fluctuation over a period of 60 days (Figure 1).

### Determination of growth attributes

The results of the current study revealed that GA_3_ alone had the potential to improve the growth parameters of the plants, i.e., root fresh weight, shoot fresh weight, root dry weight, and shoot dry weight under multiple stress conditions of HCB toxicity, and temperature stress. All these growth credentials were enhanced 1.7 to 1.9 times after GA_3_ treatment. However, the most interesting observation was the positive support of BioClay toward plant growth. Plant growth parameters were elevated by 1.1 to 1.3 times due to BioClay application. The growth regulator GA_3_ enabled *Brassica* plants to overcome HCB toxicity by introducing pollutant tolerance in them. Besides, it also elevated the plant growth parameters up to 150%. However, BioClay could enhance the plant growth up to 120% primed with HCB. The maximum growth enhancement (241%) was recorded in the plants exposed to BioClay_*GA*_, and it could be reduced up to 211% under HCB toxicity. On the other side, the toxic effects of HCB caused a 50-57% reduction in growth attributes and leaf area of the plants as compared to the control treatment. GA_3_, bioclay, and BioClay_*GA*_ proved effective in augmenting chlorophyll contents in *Brassica* plants. However, enhancement in chlorophyll contents was documented at 129% by GA_3_ application, 118% by BioClay treatment, and 143% by BioClay_*GA*_. HCB stresses plants also showed a significant increase of 138% in chlorophyll contents when primed with BioClay_*GA*_. Extreme temperature fluctuations from 25°C have deleterious effects on *Brassica* plants, retarding 15% of growth attributes, 18% of leaf area, and 14% of chlorophyll contents (Supplementary Figure 1).

### Effect leaf relative water content

The results depicted the transposed effects of extreme temperatures and HCB on *LRWC* (43.55%, and 15.6%, respectively) as compared to control *Brassica* plants. Considering the temperature gradient, the maximum *LRWC* was recorded at 25 and 35°C. However, the GA_3_ treatment increased the value of *LRWC* (40.08%) as compared to the control. BioClay treatment also enhanced 32.41% *LRWC* when applied alone, while a 46.76% increment in *LRWC* was observed under BioClay_*GA*_ treatment. The HCB treatment reduced *Pn* up to 47.62%. However, elevation in *Pn* due to GA_3_ and BioClay was 22.75%, and 18.96%, respectively. The BioClayGA application recorded the maximum Pn increase of 34.26%. The individual effect of BioClay and GA3 was found in favor of photosystem quenching *Fv/Fm* significantly higher than the effect of BioClay (20.62% increment). The most promising treatment providing the maximum increase in stomatal conductance, *Ci*, and transpiration rate were BioClay_*GA*_. However, the stress caused by extreme temperatures and HCB toxicity negatively impacted all these physiological parameters. Both the growth regulator and BioClay positively influenced the cell physiology of *B. alboglabra* (Figure 1).

### Determination of photosynthetic pigments and soluble sugars

Hexachlorobenzene toxicity significantly reduced the photosynthetic pigments up to 42.95% in comparison to the control treatment. An increase of 1.29 times was observed by GA3 and 1.18 times by BioClay treatment. BioClay_*GA*_ treatment provided more support toward total chlorophyll contents, augmenting them upto 240.94%. Low-temperature stress (0°C) caused a 14.77% chlorophyll reduction in *Brassica* plants, while 3.02% of chlorophyll contents were reduced due to high-temperature treatment (50°C). HCB signposted a significant decrease in the soluble sugar content of 3.15 mg g^–1^ as compared to 5.52 mg g^–1^ of control treatment. GA3 (6.73 mg g^–1^) and BioClay treatment (6.07 mg g^–1^) recorded significant mitigation of the sugar content. Whereas, BioClay_*GA*_ enhanced soluble sugar contents up to (7.62 mg g^–1^) in the presence of HCB, while the contents were raised upto 7.89 mg g^–1^ in the absence of toxic HCB. Both low and high-temperature stress exerted deleterious effects on *Brassica* plants in terms of soluble sugars. However, BioClay_*GA*_ mg g^–1^ were recorded at 0°C treatment (Table 1 and Figure 4).

**TABLE 1:** Effect of GA_3_ on chlorophyll *a*, chlorophyll *b*, total chlorophyll, and soluble sugars of *Brassica alboglabra* under temperature stress, and hexachlorobenzene (HCB) toxicity.

Treatments	Chl*a*	Chl*b*	Total chlorophyll	Soluble sugars (mg g^–1^)
Control	0.98 ± 0.05fg	0.51 ± 0.03g	1.49 ± 0.11fg	5.52 ± 0.14g
H	0.56 ± 0.03h	0.29 ± 0.04h	0.85 ± 0.13h	3.15 ± 0.14h
G	1.64 ± 0.06de	0.85 ± 0.04de	2.49 ± 0.07de	7.12 ± 0.13de
B	1.23 ± 0.07f	0.64 ± 0.02f	1.88 ± 0.11f	6.51 ± 0.15f
BG	2.36 ± 0.04a	1.23 ± 0.06a	3.59 ± 0.14a	7.89 ± 0.21a
HG	1.35 ± 0.06ef	0.70 ± 0.02ef	2.06 ± 0.09ef	6.73 ± 0.19ef
HB	1.10 ± 0.02fg	0.57 ± 0.03fg	1.67 ± 0.08fg	6.07 ± 0.15fg
HBG	2.07 ± 0.08b	1.08 ± 0.02b	3.14 ± 0.05b	7.62 ± 0.24b
HBG.T0	0.83 ± 0.03gh	0.43 ± 0.04gh	1.27 ± 0.05gh	4.69 ± 0.19gh
HBG.T05	1.10 ± 0.06fg	0.57 ± 0.02fg	1.67 ± 0.05fg	6.29 ± 0.13fg
HBG.T15	1.80 ± 0.02b	0.94 ± 0.04b	2.74 ± 0.05b	6.57 ± 0.22b
HBG.T35	1.99 ± 0.07bc	1.04 ± 0.02bc	3.02 ± 0.05c	7.34 ± 0.24c
HBG.T45	1.69 ± 0.05cd	0.88 ± 0.03d	2.56 ± 0.06cd	5.80 ± 0.13d
HBG.T50	0.96 ± 0.06fg	0.50 ± 0.03g	1.46 ± 0.02fg	4.75 ± 0.18g

Values demonstrate means ± SD (*n* = 5). Different letters indicate a significant difference among the treatments (*P* ≤ 0.05). C = control; H = HCB, G = GA_3_, B = BioClay, T0 = 0°C, T05 = 05°C, T15 = 15°C, T35 = 35°C, T45 = 45°C, T50 = 50°C. BG treatment was the BioClay doped with GA_3_ (BiClay*_GA_*). Duration of the temperature treatments was 2 h in the middle of light hours. However, the rest of the experiment was carried out at a constant temperature of 25°C.

**FIGURE 2:**
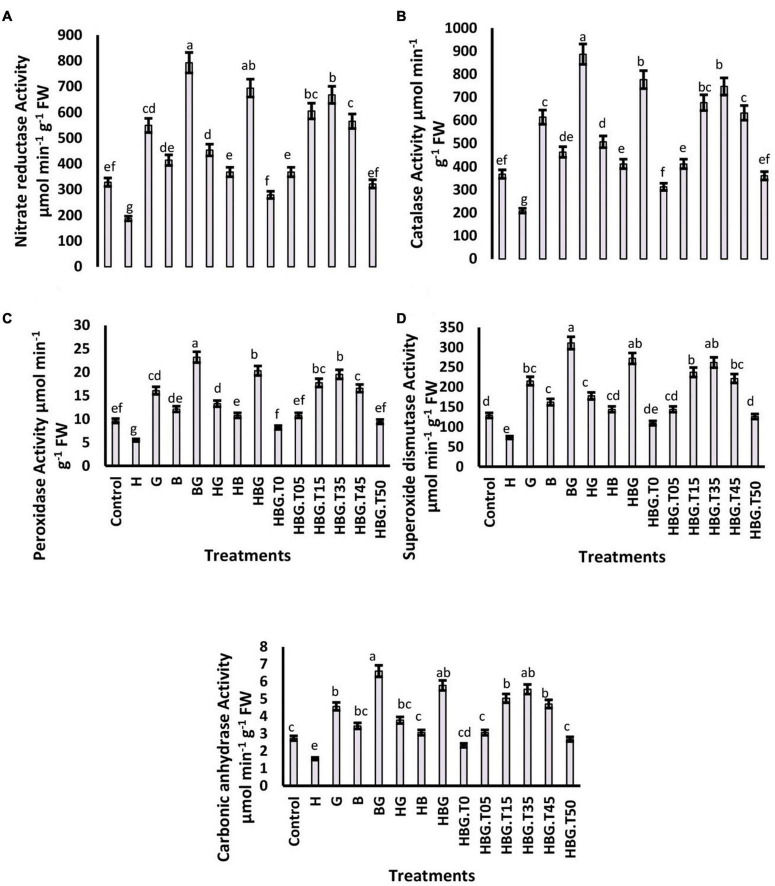
Effect of GA_3_ on antioxidant defense parameters of *Brassica alboglabra* under HCB toxicity, and temperature stress. Nitrate Reductase Activity **(A)**, Catalase Activity **(B)**, Peroxidase Activity **(C)**, and Superoxide Dismutase Activity **(D)**. Values demonstrate means ± SD (*n* = 5). Different letters indicate a significant difference among the treatments (*P* ≤ 0.05). H = HCB; G = GA_3_, B = Bioclay, T0 = 0°C, T05 = 05°C, T15 = 15°C, T35 = 35°C, T45 = 45°C, T50 = 50°C. All treatments without temperature tags got a continuous incubation temperature of 25°C.

**FIGURE 3:**
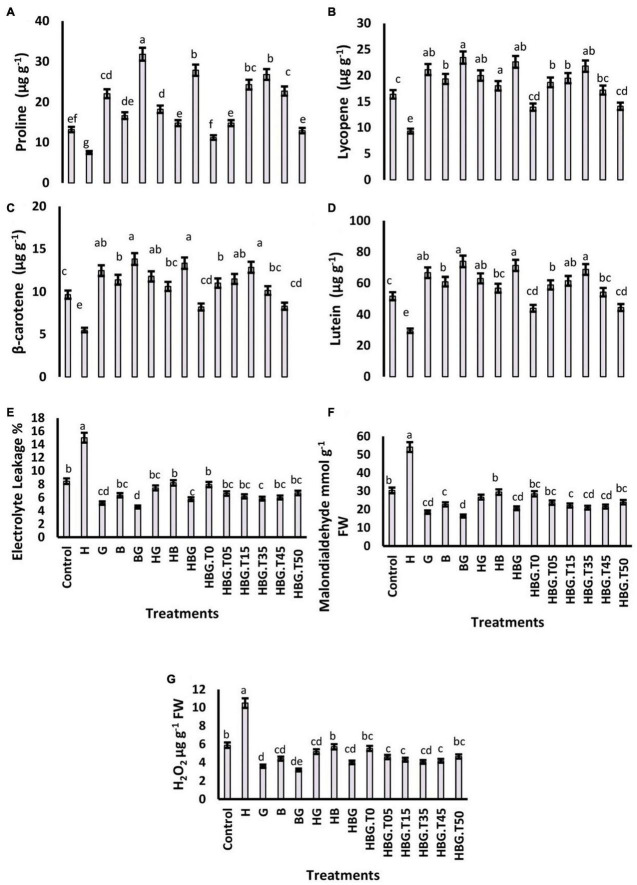
Effect of GA_3_ on physiological parameters of *Brassica alboglabra* under HCB toxicity, and temperature stress. Proline content **(A)**, Lycopene content **(B)**, β-carotene content **(C)**, and Lutein content **(D)**, Electrolyte leakage **(E)**, Malondialdehyde content **(F)**, and H_2_O_2_ content **(G)**. Values demonstrate means ± SD (*n* = 5). Different letters indicate a significant difference among the treatments (*P* ≤ 0.05). H = HCB; G = GA_3_, B = Bioclay, T0 = 0°C, T05 = 05°C, T15 = 15°C, T35 = 35°C, T45 = 45°C, T50 = 50°C. All treatments without temperature tags got a continuous incubation temperature of 25°C.

**FIGURE 4:**
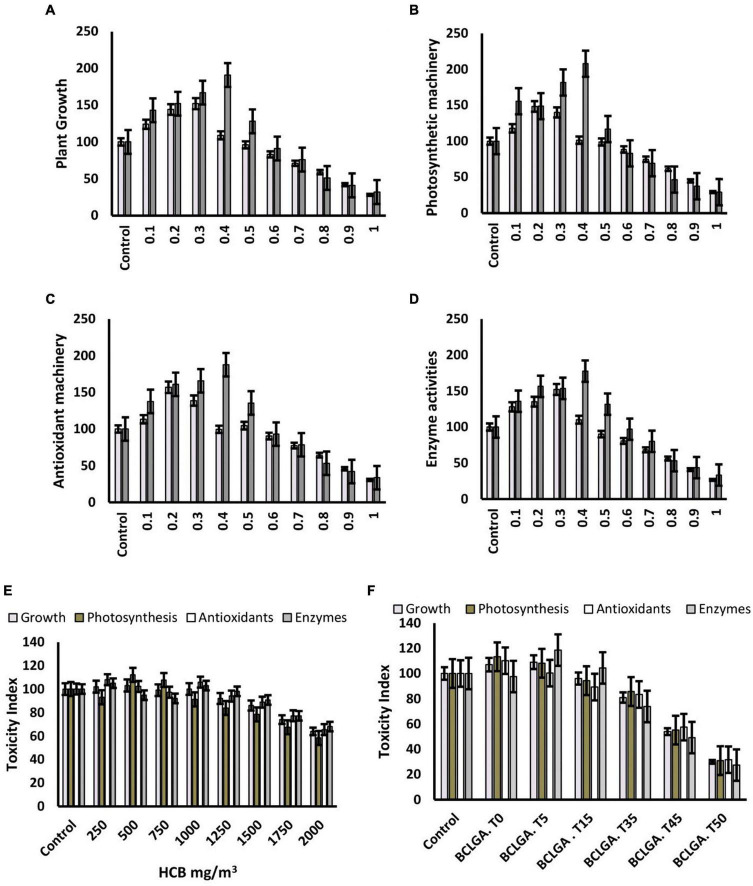
Nanotoxicity assessment of bioclay nanoparticles alone and in combination with gibberellic acid on *Brassica alboglabra* plant growth **(A)**, photosynthesis **(B)**, antioxidant machinery **(C)**, and enzyme activities **(D)**. The relation of nanotoxicity of bioclay under ambined application with gibberellic acid with HCB **(E)**, and temperature **(F)**. Values demonstrate means ± SD (*n* = 5). Different letters indicate a significant difference among the treatments (*P* ≤ 0.05). T0 = 0°C, T05 = 05°C, T15 = 15°C, T35 = 35°C, T45 = 45°C, T50 = 50°C. All treatments without temperature tags got a continuous incubation temperature of 25°C.

### Antioxidant defenses

The activity of nitrate reductase was significantly reduced by toxic HCB contents, and elevated by the application of GA_3_ and bioclay. The most effective mitigation was resulted by BioClay_*GA*_, which elevated the enzyme activity by 238.4%. The extreme temperatures adversely affected the enzyme activity, and BioClay_*GA*_ significantly mitigated temperature stress and HCB toxicity resulting in a 209.92% increase in nitrate reductase activity. Similar trends were observed in the case of other enzymes studied catalase, peroxidase, superoxide dismutase, and carbonic anhydrase. The activities of the enzymes were negatively affected by HCB toxicity and temperature fluctuations. Still, BioClay_*GA*_ successfully alleviated the hazardous effects and elevated the enzyme activities up to 216%, strengthening the antioxidant defense of the plant (Figures 2, 4).

### Enzymatic analysis

A significant increasing effect of GA_3_ was recorded in the enzymatic contents of monodehydroascorbate reductase (*MDHAR*), and dehydroascorbate reductase (*DHAR*), glutathione-s-transferase (*GST*), glutathione peroxidase (*GPX*), glutathione reductase (*GR*), and ascorbate peroxidase (*APX*) in *B. alboglabra*. BioClay treatment alone also enhanced the enzyme activities, but the extent of upregulation was significantly lower than GA_3_. The quantities of all these enzymes shared a similar trend of being elevated after BioClay and GA_3_ exposure and being reduced under toxic and stress conditions of fluctuated temperatures and the toxic environment of HCB. However, the mitigation role of BioClay_*GA*_ was dominant and elevated all the tested stress factors (Table 2 and Figure 4).

**TABLE 2:** Effect of GA_3_ on dehydroascorbate reductase (DHAR), monodehydroascorbate reductase (MDHAR), glutathione-s-transferase (GST), glutathione reductase (GR), glutathione peroxidase (GPX), and ascorbate peroxidase (APX), in *Brassica alboglabra* under temperature stress, and hexachlorobenzebe (HCB) toxicity.

Treatments	DHAR (nmol min^–1^ mg^–1^ protein)	MDHAR (nmol min^–1^ mg^–1^ protein)	GST (nmol g^–1^ FW)	GR (EU mg^–1^ protein)	GPX (μ mol g^–1^ FW)	APX (EU mg^–1^ protein)
Control	103 ± 1.9fg	60 ± 2.6c	38.1 ± 1.2fg	4.12 ± 0.1g	53.2 ± 0.8fg	2.16 ± 0.1g
H	58.71 ± 3.4i	34.2 ± 3.1e	21.717 ± 1.1h	2.35 ± 1.0h	30.32 ± 1.0h	1.2 ± 0.03h
G	132.87 ± 2.9c	77.40 ± 3.1ab	63.63 ± 1.2cd	6.88 ± 0.1de	68.62 ± 1.9de	3.61 ± 0.1de
B	121.54 ± 3.7d	70.80 ± 1.8b	48.01 ± 1.6e	5.19 ± 0.1f	62.77 ± 0.9f	2.72 ± 0.1f
BG	147.29 ± 4.1a	85.80 ± 1.9a	91.82 ± 1.5a	9.93 ± 0.2a	76.07 ± 2.2a	5.21 ± 0.2a
HG	125.66 ± 3.3cd	73.20 ± 2.2ab	52.58 ± 0.de	5.69 ± 0.2ef	64.9 ± 1.8ef	2.98 ± 0.1ef
HB	113.30 ± 3.6de	66.00 ± 1.7bc	42.67 ± 0.9f	4.61 ± 0.1fg	58.52 ± 1.7fg	2.42 ± 0.1fg
HBG	142.14 ± 5.7ab	82.80 ± 1.3a	80.39 ± 2.1b	8.69 ± 0.2b	73.42 ± 1.5b	4.56 ± 0.1b
HBG.T0	87.55 ± 2.8h	51.00 ± 2.7d	32.39 ± 1.1g	3.50 ± 0.1gh	45.22 ± 1.3gh	1.83 ± 0.1gh
HBG.T05	117.42 ± 2.6de	68.40 ± 1.1bc	42.67 ± 2.1f	4.61 ± 0.2fg	60.65 ± 1.4fg	2.42 ± 0.1fg
HBG.T15	122.57 ± 3.6d	71.40 ± 0.9b	70.10 ± 1.5c	7.58 ± 0.3b	63.31 ± 2.0b	3.97 ± 0.1b
HBG.T35	136.99 ± 4.1bc	79.80 ± 2.5ab	77.34 ± 2.1bc	8.36 ± 0.2c	70.76 ± 1.6c	4.38 ± 0.2bc
HBG.T45	108.15 ± 2.4f	63 ± 1.6bc	65.53 ± 2.4cd	7.09 ± 0.2d	55.86 ± 1.3cd	3.72 ± 0.1d
HBG.T50	88.58 ± 2.6h	51.6 ± 1.1d	37.34 ± 1.2fg	4.04 ± 0.1g	45.75 ± 1.7fg	2.12 ± 0.1g

Values demonstrate means ± SD (n = 5). Different letters indicate a significant difference among the treatments (*P* ≤ 0.05). C = control; H = HCB, G = GA_3_, B = BioClay, T0 = 0°C, T05 = 05°C, T15 = 15°C, T35 = 35°C, T45 = 45°C, T50 = 50°C. BG treatment was the BioClay doped with GA_3_ (BioClay*_GA_*). Duration of the temperature treatments was 2 hours in the middle of light hours. However, the rest of the experiment was carried out at a constant temperature of 25°C.

### Proline, lycopene, β-carotene and lutein contents

Results showed that proline contents were significantly retarded (35.21%) in the plants exposed to HCB. On the other side, GA_3_ increased proline contents 1.39 times as compared to the control treatment. BioClay treatment also exhibited positive effects on proline contents comparable with GA_3_. Lycopene, β-carotene, and lutein were also negatively regulated by the temperature extremes (low and high temperatures). However, the three treatments, i.e., GA_3_, BioClay, and BioClay_*GA*_, significantly enhanced all these biochemicals (Figure 3).

### Electrolyte leakage MDA and H_2_O_2_

The *EL* was increased by HCB treatment and extreme temperatures, either low or high temperatures. An increase of 210.36% was recorded in *EL* due to HCB. However, the increased *EL* due to extreme temperatures was non-significant as compared to the control treatment. MDA was also increased by two times under the toxic influence of HCB. A similar trend was followed by H_2_O_2_ contents, which were elevated by toxic HCB and extreme temperatures (215.37 and 10.23%, respectively). Plant growth regulator GA_3_ and BioClay treatments successfully downregulated H_2_O_2_ contents reducing the risk of oxidative damage to plant cells (Figure 3).

### Water potential, leaf osmotic potential, and membrane stability index

Exogenously applied GA_3_ caused a 128.57% enhancement in the leaf water potential of *B. alboglabra*. However, plants grown in HCB toxic environment contained 57.14% less leaf water potential. Similarly, extreme temperatures also adversely affected leaf water potential. Whereas, significant enhancement in leaf water potential and mitigation of HCB toxicity were observed by BioClay and BioClay_*GA*_ treatments. A similar trend was found in leaf osmotic potential and membrane stability index (MSI). BioClay_*GA*_ successfully mitigated the stress effects of extreme temperatures and HCB toxicity (Table 3).

**TABLE 3:** Effect of GA_3_ and BioClay nanosheets on water potential, leaf osmotic potential, membrane stability index (MSI), root, shoot, leaf and seed HCB uptake, translocation factor (TF), avoidance efficacy (AE) and pollutant tolerance index (PTI) of *Brassica alboglabra* under temperature stress, and hexachlorobenzebe (HCB) toxicity.

Treatments	Water potential	Leaf osmotic potential	MSI	Root HCB (μ g g^–1^)	Shoot HCB (μ g g^–1^)	Leaf HCB (μ g g^–1^)	Seed HCB (μ g g^–1^)	TF	AE (%)	PTI
Control	0.63 ± 0.01d	0.88 ± 0.03d	0.93 ± 0.02d	-	-	-	-	-	-	-
H	0.36 ± 0.1f	0.50 ± 0.05f	0.53 ± 0.01f	89.00 ± 0.3a	2.67 ± 0.03a	2.33 ± 0.1a	1.96 ± 0.2a	1.32 ± 0.02a	2.68h	33.63 ± 1.8g
G	0.81 ± 0.01b	1.14 ± 0.03b	1.20 ± 0.02b	-	-	-	-	-	-	-
B	0.74 ± 0.02c	1.04 ± 0.02bc	1.10 ± 0.02c	-	-	-	-	-	-	-
BG	0.90 ± 0.02a	1.26 ± 0.01a	1.33 ± 0.02a	-	-	-	-	-	-	-
HG	0.77 ± 0.01bc	1.07 ± 0.01bc	1.13 ± 0.04bc	44.00 ± 0.5bc	1.32 ± 0.06bc	1.15 ± 0.07c	0.97 ± 0.03c	0.65 ± 0.07bc	6.58 ± 0.08e	81.42 ± 0.05d
HB	0.69 ± 0.02cd	0.97 ± 0.02cd	1.02 ± 0.05cd	48.50 ± 0.2b	1.46 ± 0.04b	1.27 ± 0.09b	1.07 ± 0.01b	0.72 ± 0.02b	5.42 ± 0.06f	66.08 ± 1.9e
HBG	0.87 ± 0.03ab	1.21 ± 0.03ab	1.28 ± 0.02ab	34.00 ± 0.1cd	1.02 ± 0.02cd	0.89 ± 0.03cd	0.75 ± 0.03cd	0.50 ± 0.02cd	10.12 ± 0.05a	124.49 ± 2.6a
HBG.T0	0.54 ± 0.01e	0.75 ± 0.01e	0.79 ± 0.03e	47.00 ± 0.2bc	1.41 ± 0.02bc	1.23 ± 0.04bc	1.03 ± 0.02bc	0.70 ± 0.01bc	4.04 ± 0.04bc	50.15 ± 1.9f
HBG.T05	0.72 ± 0.02c	1.00 ± 0.02c	1.06 ± 0.02c	39.00 ± 0.3c	1.17 ± 0.03c	1.02 ± 0.03d	0.86 ± 0.01d	0.58 ± 0.01d	5.41 ± 0.05f	66.08 ± 2.0e
HBG.T15	0.75 ± 0.01bc	1.05 ± 0.01bc	1.11 ± 0.01bc	36.50 ± 0.4cd	1.10 ± 0.01cd	0.96 ± 0.01de	0.80 ± 0.01de	0.54 ± 0.02de	8.82 ± 0.07c	108.56 ± 3.3b
HBG.T35	0.84 ± 0.03ab	1.17 ± 0.01ab	1.24 ± 0.02ab	34.50 ± 0.3cd	1.04 ± 0.04cd	0.90 ± 0.02e	0.76 ± 0.02e	0.51 ± 0.02de	9.72 ± 0.09b	119.77 ± 4.1ab
HBG.T45	0.66 ± 0.02cd	0.92 ± 0.01cd	0.98 ± 0.02cd	35.50 ± 0.4cd	1.07 ± 0.02cd	0.93 ± 0.01de	0.78 ± 0.03de	0.53 ± 0.02de	8.25 ± 0.04d	101.48 ± 2.1bc
HBG.T50	0.54 ± 0.02e	0.76 ± 0.01e	0.80 ± 0.01e	39.50 ± 0.4c	1.19 ± 0.03c	1.03 ± 0.02cd	0.87 ± 0.02d	0.58 ± 0.01d	4.73 ± 0.06g	57.82 ± 2.0ef

Values demonstrate means ± SD (*n* = 5). Different letters indicate a significant difference among the treatments (*P* ≤ 0.05). C = control; H = HCB, G = GA_3_, B = BioClay, T0 = 0°C, T05 = 05°C, T15 = 15°C, T35 = 35°C, T45 = 45°C, T50 = 50°C. BG treatment was the BioClay doped with GA_3_ (BioClay*_GA_*). Duration of the temperature treatments was 2 h in the middle of light hours. However, rest of the experiment was carried out at a constant temperature of 25°C.

### Hexachlorobenzene contents, translocation factor and pollutant tolerance index

During the current study, the HCB accumulation was recorded higher in roots (89.0 μg g^–1^) as compared to shoots (2.67 μg g^–1^) and leaves (2.33). An interesting observation was of 1.96 μg g^–1^ HCB contents in the *Brassica* seeds. In extreme temperatures, especially the low temperature, was HCB accumulations enhancer facilitating HCB aero-concentration in the plant tissues as well as in seeds (0.86 μg g^–1^). Both the GA_3_ and BioClay significantly reduced the deleterious effect of HCB, and extreme temperatures lowering down the HCB accumulations in *Brassica* roots, shoots, leaves, and seeds. The most promising stress alleviation was inherited by BioClay_*GA*_ treatment resulting in 0.75 μg g^–1^ HCB concentration in *Brassica* seeds. Three treatments, GA_3_, BioClay, and BioClay_*GA*_, bear an optimistic activity with respect to the *TF* of HCB. *Brassica* plants primed with GA_3_ showed a *TF* value of 0.65 in comparison to the 1.32 *TF* value of the control treatment. The minimum translocation of HCB (0.5) occurred in BioClay_*GA*_ treated plants. Pollutant tolerance index in BioClay_*GA*_ treated plants was also recorded at the maximum (124.49), followed by GA_3_ (81.42) and BioClay (66.08). However, extreme temperatures have detrimental effects on plants’ ability to tolerate the pollutant. Similarly, the plants’ avoidance efficacy (AE) was negatively impacted by the temperature fluctuations, whereas BioClay, GA_3_, and BioClay_*GA*_ elevated the avoidance of the plants against HCB (Table 3 and Figure 4).

## Discussion

For the first time, the present investigation sets a procedure for sustainable exogenous delivery of GA_3_ to a plant surface through impregnating with bioclay. GA_3_ is a plant hormone that is mainly responsible for cell elongation, internodal length, and defining plant height (Montague, 1993). The plant response toward GA_3_ is truly dose and duration dependent, making the exogenous application of GA3 to attain the desired results (Chandra et al., 2019). In this way, sustained availability of the fixed hormonal portion is essential to involve GA_3_ in field conditions to get the wanted results. The present research makes it simpler to support the GA_3_ portion of the plants during longer development periods by utilizing BioClay impregnated with GA_3_. Besides, GA_3_ bears the propensity to tune the genetic controls of the plant. The growth hormone is engaged with initializing the expression of starch and sugar-related h genetic cascade, which at last characterizes the plant’s energy economy (Chandra et al., 2019). This property of the phytohormone has been utilized to harmonize the physiological balances during stress conditions.

The stress factors (e.g., HCB) incite physiological imbalances and promote *EL*, and oxidative stress, leading to adaptive cellular responses in the form of solute accumulation (Shaddad et al., 2013). This establishes a groundwork for the expanded air fixation coefficient of HCB in various tissues of *Brassica* plants. Furthermore, the study first time decides the movement variable of HCB in B. alboglabra plant tissues, expounding the destiny of the toxin when consumed in the eatable plant. Additionally, the scientific investigation explored the mitigation behavior of BioClay, and GA_3_ by reharmonization of the stress indicators, e.g., *EL*, *LRWC*, gaseous exchange, antioxidant defenses, and enzymes’ activities. *Ci*, *EL*, *Pn*, leaf water potential, etc. are the main tools of environmental toxicants to retard plant growth and disturb metabolism (Khan et al., 2017; Yasin et al., 2018; Li et al., 2021). The study recommends BioClay_*GA*_ as a resilient stress alleviator that can reharmonize the plant metabolism and rehabilitate plant cell homeostasis. The current study reports the BioClay_*GA*_ as an effective stress alleviator that elevates the *Pn*, photosynthetic pigments (Chl*a*, and Chl*b*), and total soluble sugars. This multitude of variables mutually restored the physiological elements and growth parameters because of work on the photosynthetic action of Brassica. Then again, temperature stress and HCB harmfulness are the significant stressors of the present climate that can shorten plan development and physiological properties. The augmented biosynthesis of chlorophyll contents positively affected the photosynthesis and growth of the plants treated by BioClay_*GA*_.

During stress conditions, the minimal objective of a plant is to make due through the shocking time, and the minimal objective of a rancher is to get sufficient respect to meet the development costs. This is achieved through the plant tolerance mechanism against stress factors (Ahmad et al., 2014b; Li et al., 2021). The study calculated the PTI for HCB-affected plants of *B. alboglabra*. Additionally, the AE of *Brassica* plants against toxic HCB was a prime finding of the current research work, which was calculated against twice the concentration of the pollutant reported harmful to the plants. Evasion is the best methodology to remediate the environmental pollutants, letting them out of the food chain for normal debasement (Waheed Ullah Khan et al., 2017). Using GA_3_ impregnated BioClay has been proved a unique technique, hampering the entry of HCB into the food chain, which would surely be detoxified by natural degradation with the passage of time.

Moreover, the alone and combined effect of BioClay with the phytohormone resulted in positive physiochemical alterations in plants. Beforehand a couple of studies involved BioClay for the effective conveyance of RNAi to get the plants safe against microorganisms (Fletcher et al., 2020). In any case, there was no extreme end in regards to the development guideline conduct of the nanosheets. The ongoing concentrate first time featured the steady job of the earth nanosheets toward plant development. It not only supported the growth parameters of *B. alboglabra* but also augmented antioxidant enzymes and other physiological factors. The study proved that the BioClay was of immense potential to be used as a plant growth enhancer under stress conditions. Having no negative cross-talk with GA_3_ is an advantageous feature of BioClay explored in this study.

*LRWC* value acts as a biomarker for plant-water relationships. High-temperature stress has negative effects on osmotic potential as well as water. Low temperature also negatively impacted *LRWC* values of *Brassica* plants, indicating reduced drought resistance (Wang et al., 2019). During current research, all the three test treatments, i.e., BioClay, GA_3_, and BioClay_*GA*_, significantly improved *LRWC* proving a good choice for low rainfall areas and areas adversely affected by global warming. Lipid peroxidation is one of the major reasons of membrane injuries, is one of the leading causes of disrupted metabolic patterns in the plant cells, and is considered a biomarker of cellular injuries for a plant under stress (Ayala et al., 2014). A majority of writing accessible fosters an association between expanded biosynthesis of H_2_O_2_ with oxidative wounds of the phones and extreme driving toward putrefaction (Ayala et al., 2014). Yet, BioClay and GA_3_ inhibited the biosynthesis of the oxidative stressor H_2_O_2_. Moreover, the utilization of BioClayGA decreased the MDA, the last result of lipid peroxidation, and demonstrated the proficiency of the treatment against natural stressors. The *EL* caused by damaged cellular membrane reduces plant growth and biomass production. A few examinations uncovered that the up-directed exercises of antioxidative hardware decline the blend of ROS in focused plants. Superoxide is detoxified into a less injurious H_2_O_2_ by the activity of *SOD*, which is subsequently converted into H_2_O through the activity of *POD*, *CAT*, and *APX* (Chen and Heuer, 2013). The exogenous utilization of BioClayGA upgraded pressure resistance by working on the action of the antioxidative framework in focused plants.Henceforward, the up-regulation in antioxidative machinery besides the decreased level of *EL*, H_2_O_2_, and MDA highlighted the favorable role of BioClay_*GA*_ in the reduction of oxidative injuries and regulation of redox homeostasis in stressed plants.

Superoxide dismutase is ranked among the primary antioxidant defenses of the plant cell, crucial to surviving under stress conditions. Although *SOD* and *CAT* are mainly localized in peroxisomes and mitochondria of the plant cells (Zaheer et al., 2018), they still showed a significant response to stress conditions. However, BioClay_*GA*_ treatment mitigated the stress physiology of *B. alboglabra*. The ongoing study gives nitty-gritty interrelation between GA_3_, bioclay, BioClayGA, H_2_O_2_, MDA, and cell reinforcements catalyst exercises. By taking into account the by and large physiological reactions of the plant, it tends to be presumed that every one of the three medicines (GA_3_, bioclay, and BioClay_*GA*_) strengthened plant defenses against environmental stresses and HCB toxicity. However, BioClay_*GA*_ was the most promising formulation in this regard. The scavenging of reactive oxygen species (ROS) is a prime function in a plant cell to thrive under stress conditions (Abbas et al., 2020). H_2_O_2_ is the main source of ROS, and ascorbate is one of the most powerful substrates for scavenging H_2_O_2_. The higher ROS biosynthesis than it is being scavenged (by the antioxidant system) leads to irreversible oxidative damages (Ahmad et al., 2014a). Ascorbate, glutathione, and carotenoids belong to the non-enzymatic antioxidant defense system. However, *DHAR*, *MDHAR*, *GST*, *GR*, *GPX*, *APX*, *SOD*, and *CAT* are key components of the enzymatic antioxidant system (Konieczny et al., 2014). BioClayGA has carried out a double role to reinforce the oxidative guard arrangement of *B. alboglabra* while lifting the movement of cell reinforcements to build the guarded layers of the plant further.

Researchers have always been interested in increasing edible plants’ proline and carotenoids (lycopene, β-carotene, and lutein) contents. Carotenoids are the pigmented elements of plants which contribute to the photosynthetic machinery and perform a protective function against photo-damage (Singh and Sahota, 2018). Foods rich in carotenoid contents protect humans from age-related diseases. Lycopene is a major carotenoid in *Brassica* with characteristic red color and special antioxidant properties (Elbadrawy and Sello, 2016). Proline is an excellent part of protein biosynthesis hardware, which characterizes the cell structure, digestion, sustenance profile, wound recuperating, antioxidative responses, and protection reactions (Wu et al., 2011). Particularly proline plays a vital role in the stability of macromolecules structure and turgidity of cellular membrane structure in stressed plants. Proline scavenges ROS and attenuates stress in plants. During the present study, BioClay_*GA*_ successfully enhanced proline and carotenoid contents in *Brassica* plants under multiple stress conditions. It hardened the plants and made them tolerant against temperature stress and HCB toxicity.

The increased HCB concentration in roots of plants growing in a POP toxic environment is because vacuole and cell walls of roots may amass and hold higher HCB contents compared to shoot cells (Dong et al., 2017). The exogenous application of BioClay_*GA*_ decreased HCB contents in plants due to modified homeostasis. Moreover, every one of the advantages of lessening HCB contents in foliage and seeds of *B. alboglabra* is the principal accomplishment of the study, which lies with the limited section of HCB in the plant. Taking into account the exchange nature, persistance, bioaccumulation, and deadly impacts of HCB, the BioClayGA treatment accomplished a sign of hindering airborne contamination from entering the human food chain. Also, BioClayGA application decreased the ACF of the Brassica shoots, obstructing the HCB move at the initial step from air to food. The study can be expanded to seek the bio-concentration conduct of different POPs in food plants.

## Conclusion

This study revealed that the combination of BioClay and GA_3_ successfully improved the plant growth and physiological performance of *B. alboglabra* under multiple stresses (hexachlorobenzene and temperature extreme). Moreover, exogenously applied BioClay_*GA*3_ ameliorated the combined stress of hexachlorobenzene and temperature extremes through decreased production of ROS, modulated synthesis of osmoregulators, and improved antioxidative system. BioClay_*GA*3_ inhibited air absorption of HCB and reduced its transfer coefficient among different plants. Accordingly, we are confident that the stress alleviation behavior of BioClay_*GA*3_ offers an innovative approach to sustainable agricultural practice for future food security.

## Data availability statement

The original contributions presented in this study are included in the article/Supplementary material, further inquiries can be directed to the corresponding author.

## Author contributions

AaA, NY, IS, SS, and WA conceived the idea, planned experiments, and managed resources for the study. SU, TK, AqA, and MY performed the experimentations, collected and anlayzed the data, and wrote the manuscript draft. AaA, NY, WA, IS, SS, SU, TK, AqA, and MY revised and finalized the manuscript. All authors contributed to the article and approved the submitted version.
