# Supplementary material for: BioClay nanosheets infused with GA3 ameliorate the combined stress of hexachlorobenzene and temperature extremes in Brassica alboglabra plants
Source: Front Plant Sci. 2022 Oct 6;13:964041. doi: 10.3389/fpls.2022.964041 (PMC9583914; doi:10.3389/fpls.2022.964041)
Supplement: Supplementary file 1 [file Data_Sheet_1.docx]

# BioClay nanosheets infused with GA3 ameliorates combined stress of hexachlorobenzene and temperature extremes in *Brassica alboglabra* plants

Tingquan Wu ^1^, Iqra Shahzadi ^2^, Sharoon Shahzad ^3^, Wang Rui ^1^, Sami Ullah ^4^, Tanveer Alam Khan ^5^, Aqeel Ahmad ^6^, Mohammad Yusuf* ^7^

^1^ Guangdong Academy of Agricultural Sciences,Guangzhou, Guangdong, China.

^2^ School of Resource and Environmental Science, Wuhan University, Wuhan 430079, China

^3^ Incharge Medical Officer, Basic Health Unit Munday key District Kasur, Pakistan.

^4^ Department Of Forestry, College of Agriculture, University Of Sargodha, Postal Code 40100 Sargodha, Pakistan

^5^ Department of Botany, Aligarh Muslim University, Uttar Pardesh, India

^6^ Institute of Geographic Sciences and Natural Resources Rersearch, Chinese Academy of Science, Beijing, China

^7^ Department of Biology, College of Science, United Arab Emirates University, Al Ain – 15551, UAE.

Corresponding Author: Mohammad Yusuf,

Email: [myusuf.alig@uaeu.ac.ae](mailto:myusuf.alig@uaeu.ac.ae);

Tel: (+971)3713 6199

**Table S1:** List of treatment combinations of HCB stress, temperature stress, and BDE-28 toxicity to *Brassica alboglabra*.

| Treatments | Description |
| --- | --- |
| Control | Standard growth solution |
| H | HCB |
| G | GA_3_ |
| B | BioClay |
| BG | BioClay_GA_ |
| HG | HCB + GA_3_ |
| HB | HCB + BioClay |
| HBG | HCB + BioClay_GA_ |
| HBG.T0 | HCB + BioClay_GA_ + 0°C (2h) |
| HBG.T05 | HCB + BioClay_GA_ + 05°C (2h) |
| HBG.T15 | HCB + BioClay_GA_ + 15°C (2h) |
| HBG.T35 | HCB + BioClay_GA_ + 35°C (2h) |
| HBG.T45 | HCB + BioClay_GA_ + 45°C (2h) |
| HBG.T50 | HCB + BioClay_GA_ + 50°C (2h) |

**Table S2:** Properties of Bioclay nanosheets.

| **Property** | **State** |
| --- | --- |
| Width | 1.7-3.2 µm |
| Thickness | 80-150 nm |
| Electron diffraction angle | 45° |
| Interfacial angle | 43.4° |
| Interplanar lattice spacing | 0.272 nm |
| Lattice spacing of atomic planes | 0.35 nm |
| Lattice fringe spacing | 0.384 nm |
| Nature | Crystalline |

f

cd

de

de

de

bc

ab

a

cd

de

bc

ab

de

de

de

bc

ab

cd

c

ab

ab

ef

de

ab

de

de

(H)

(G)

(E)

(C)

de

cd

cd

d

d

de

cd

cd

bc

e

ab

a

cd

cd

b

ab

b

cd

b

b

a

de

b

de

de

ab

e

b

a

ab

b

a

cd

b

c

b

ef

d

e

bc

a

d

c

e

c

(F)

b

ef

d

cd

f

e

c

c

d

c

bc

a

bc

de

cd

e

b

bc

d

f

a

cd

e

(A)

(B)

(D)

d

ab

a

c

f

de

bc

e

d

b

cd

c

f

b

a

c

c

c

**Figure S1:** Effect of HCB on growth attributes of *Brassica alboglabra* under Cd stress, temperature stress, and BDE-28 toxicity. Root length (A), Shoot length (B), Root mass (C), Shoot mass (D), Leaf area (E), SPAD chlorophyll value *Y*:0.78*X*+19.7 (F). Values demonstrate means ± SD (n=5). Different letters indicate significant difference among the treatments (*P*≤0.05). H=HCB; G=GA_3_, B=Bioclay, T0=0°C, T05=05°C, T15=15°C, T35=35°C, T45=45°C, T50=50°C. All treatments without temperature tags got a continuous incubation temperature of 25°C.
